# Fate of a Deep Eutectic Solvent upon Cosolvent Addition: Choline Chloride–Sesamol 1:3 Mixtures with Methanol

**DOI:** 10.1021/acssuschemeng.1c03809

**Published:** 2021-09-02

**Authors:** Matteo Busato, Alessandra Del Giudice, Valerio Di Lisio, Pierpaolo Tomai, Valentina Migliorati, Alessandra Gentili, Andrea Martinelli, Paola D’Angelo

**Affiliations:** Department of Chemistry, University of Rome ”La Sapienza”, P.le A. Moro 5, 00185 Rome, Italy.

**Keywords:** deep eutectic solvents, choline chloride, sesamol, methanol, FTIR spectroscopy, molecular dynamics, X-ray scattering, low-transition temperature mixtures

## Abstract

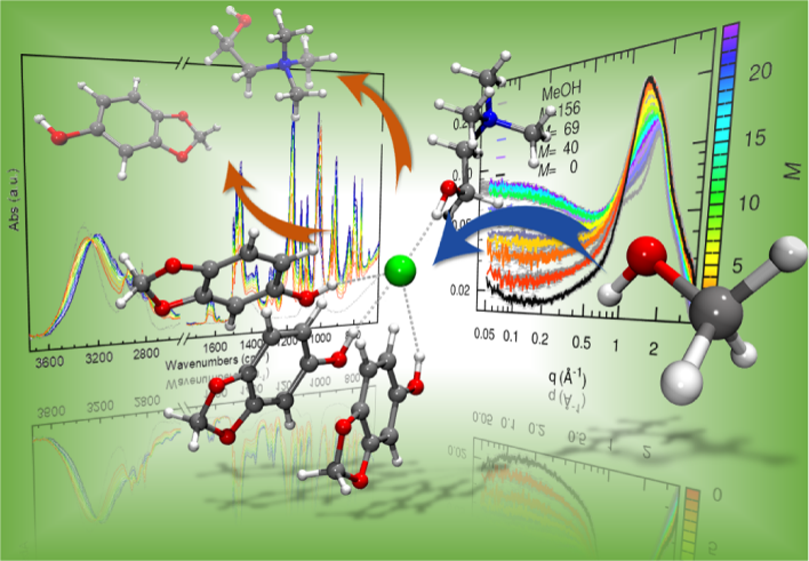

The changes upon methanol (MeOH) addition in the structural arrangement of the highly eco-friendly deep eutectic solvent (DES) formed by choline chloride (ChCl) and sesamol in 1:3 molar ratio have been studied by means of attenuated total reflection Fourier transform infrared spectroscopy, small- and wide-angle X-ray scattering (SWAXS), and molecular dynamics simulations. The introduction of MeOH into the DES promotes the increase of the number of Cl–MeOH hydrogen bonds (HBs) through the replacement of sesamol and choline molecules from the chloride anion coordination sphere. This effect does not promote the sesamol–sesamol, choline–choline, and sesamol–choline interactions, which remain as negligible as in the pure DES. Differently, the displaced sesamol and choline molecules are solvated by MeOH, which also forms HBs with other MeOH molecules, so that the system arranges itself to keep the overall amount of HBs maximized. SWAXS measurements show that this mechanism is predominant up to MeOH/DES molar ratios of 20–24, while after this ratio value, the scattering profile is progressively diluted in the cosolvent background and decreases toward the signal of pure MeOH. The ability of MeOH to interplay with all of the DES components produces mixtures with neither segregation of the components at nanoscale lengths nor macroscopic phase separation even for high MeOH contents. These findings have important implications for application purposes since the understanding of the pseudophase aggregates formed by a DES with a dispersing cosolvent can help in addressing an efficient extraction procedure.

## Abstract 2

The structural changes of a sustainable deep eutectic solvent formed by choline chloride and sesamol upon methanol addition as a cosolvent have been studied.

## Introduction

Deep eutectic solvents (DESs) are gaining increasing attention as a more sustainable alternative to traditional organic solvents for several applications.^1,2^ DESs are a compositionally heterogeneous class of solvents formed by both molecular and ionic compounds acting as hydrogen bond donors (HBDs) and hydrogen bond acceptors (HBAs), very often based on quaternary-ammonium salts such as choline chloride (ChCl) and HBDs such as amines, amides, carboxylic acids, and alcohols.^3,4^ The combination of the HBA and HBD in proper proportions gives rise to a eutectic with a melting point in the phase diagram that is lower than those of the individual components. The origins of such behavior have been long debated and rely on the extensive hydrogen bond (HB) network that is established among the components upon the melting process so that the system is usually arranged to maximize the molecular interactions.^5−8^ Once discovered, DESs suddenly gained much attention owing to some outstanding properties like negligible vapor pressure, nonflammability, high conductivity, high solvation ability, and low toxicity.^9^ The term “natural deep eutectic solvents” (NADES) was also coined to describe a subset of DESs obtained by components that are primary metabolites of living cells, like amino acids, organic acids, sugars, and choline derivatives, dramatically increasing the biocompatibility and eco-friendliness of these materials.^10−12^ DESs also show an intrinsic nature of “designer solvents”, since the chemical nature of the constituents can be tailored to meet desired chemical–physical requirements.^9,13^

Besides the study of “pure” DESs, interest has recently been devoted to DES mixtures formed upon cosolvent addition.^14−22^ Indeed, the addition of molecular solvents such as water, alcohols, or alkanes, has been shown to dramatically affect several DES key properties like density, viscosity, conductivity, CO_2_ solubility, and enzyme activity,^23−28^ eventually providing eutectics with enhanced performances and lower costs.^22,29^ Cosolvent addition, therefore, represents a further designing strategy that is often less resource-demanding instead of acting on the chemical nature of the DES constituents, allowing optimization within the field of green chemistry, waste reduction, and E-factor improvement.^30,31^ Moreover, due to their usually high viscosity, DESs are often diluted with dispersing agents in extraction applications.^13,32,33^ In this framework, providing a fundamental understanding of the intermolecular interactions occurring in DES mixtures with cosolvents will facilitate the design of task-specific media addressing new technological and environmental requirements.^34^

**Figure 1:**
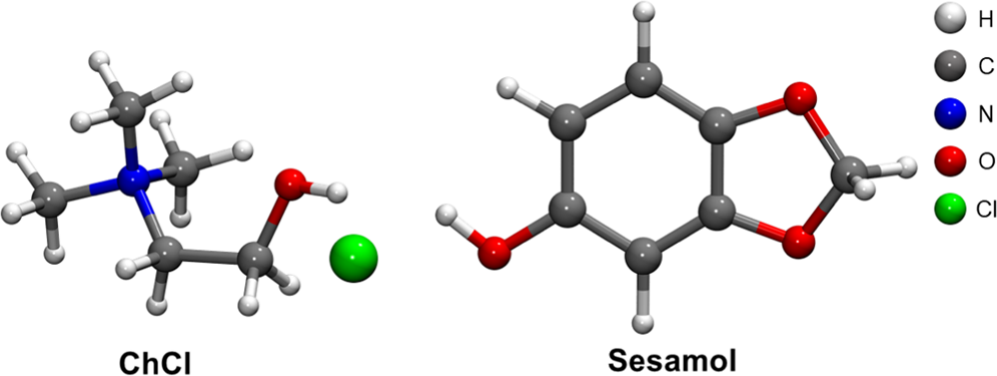
Molecular structure of the ChCl/sesamol 1:3 DES components: choline chloride (ChCl) and sesamol. The atoms are represented according to the color code on the top-right.

Here, we present a study on the structural modifications of a DES formed by ChCl and sesamol (Figure 1) in the 1:3 molar ratio upon methanol (MeOH) addition. The quasihydrophobic ChCl/sesamol 1:3 DES has been only recently developed and showed outstanding results for what concerns liquid–liquid microextraction applications in food and biological samples,^15,32,33,35−37^ besides an intrinsically eco-friendly nature provided by the peculiarities of its components.^32,33,35^ In fact, ChCl is known to be a low-price nontoxic compound, while sesamol is a powerful antioxidant able to prevent the spoilage of oils and presents chemoprotective, antimutagenic, and antihepatotoxic properties.^38,39^ In this light, this mixture can be rightfully settled in the realm of the “NADES” family. Technically, the ChCl/sesamol 1:3 eutectic can be classified as a low-transition temperature mixture (LTTM)^9,13^ since crystallization has not been experimentally observed; however, the terminology “DES” will be used herein. MeOH has been chosen as the prototypical alcohol since alcohols with different chain lengths are often employed as DES dispersive agents in liquid–liquid microextractions.^13,32,33,40^ In addition, the hydroxyl group of MeOH enables the formation of an intricate HB network resembling that formed by water, but the contemporary presence of the methyl group also allows one to gain insights into the interactions of the studied system with an organic species.^41,42^ To shed light on the changes in the interactions occurring in solution upon MeOH addition, MeOH/ChCl/sesamol mixtures with different *M*:1:3 molar ratios have been studied by means of attenuated total reflection Fourier transform infrared (ATR-FTIR) spectroscopy, small- and wide-angle X-ray scattering (SWAXS) measurements, and molecular dynamics (MD) simulations. We expect that the obtained fundamental insights will provide knowledge on the link between the structural arrangement of these systems and their macroscopic physical–chemical properties, being a step forward in the development of new DES mixtures and ultimately on their possible innovative applications.

## Materials and Methods

### Sample Preparation

ChCl (≥99%), sesamol (≥99%), and MeOH (HPLC grade) were purchased from Aldrich-Fluka-Sigma S.r.l. Sesamol and ChCl were dried separately at 50 °C for 24 h and at 100 °C for 16 h in a muffle oven to remove water traces. The removal of water was checked by analyzing the dried components via thermogravimetric analysis (TGA) (METTLER TOLEDO TG50, measuring module linked to a METTLER TOLEDO TC 10 interface). Ten milligrams of each dried sample was weighed in a ceramic pan, which, after being closed with a lid, was rapidly placed in the measuring furnace and purged with 30 mL min^–1^ nitrogen flux. TGA curves were acquired during the heating from 30 to 500 °C at 10 °C min^–1^. The ChCl/sesamol 1:3 DES was then prepared in a weighing bottle by mixing the pure constituents in the required molar ratio and heating at 60 °C until a homogeneous and amber viscous liquid was obtained (20 min). This DES was used to prepare MeOH/ChCl/sesamol mixtures at *M*:1:3 ratios with *M* values in the 1–156 range. The full list of the prepared samples with their relative compositions and densities is reported in Table S1.

### ATR-FTIR Spectroscopy

Infrared spectra were collected on pure MeOH and on MeOH/ChCl/sesamol mixtures at various *M*:1:3 molar ratios with a Nicolet 6700 FTIR spectrometer equipped with a Specac Golden Gate ATR accessory. Absorbance spectra were collected in the 4000–650 cm^–1^ range, by coadding 200 scans at 4 cm^–1^ resolution. Liquid samples were deposited on the ATR diamond crystal and closed hermetically to avoid evaporation during data acquisition. Two spectral intensities were recorded for each sample at 3189 and 3309 cm^–1^ using a two-point linear baseline between 3800 and 2560 cm^–1^. Moreover, the area of the 765 cm^–1^ band was calculated by spectral integration between 775 and 748 cm^–1^.

### Molecular Dynamics Simulations

Classical MD simulations have been performed on MeOH/ChCl/sesamol systems at different *M*:1:3 molar ratios. Cubic boxes were built with ∼100 Å side lengths and a number of species chosen to reproduce the density of each mixture (Table S2). Structures and interactions of the MeOH and sesamol molecules were represented with the OPLS-AA force field,^43^ while OPLS-compatible parameters developed by Canongia Lopes and Padua were employed for the choline cation^44^ and for the chloride anion.^45^ Cross-terms for the Lennard-Jones interactions were constructed with the Lorentz–Berthelot combining rules. A cutoff radius of 12 Å was employed for all nonbonded interactions, while long-range electrostatic forces were taken into account with the particle mesh Ewald (PME) method.^46,47^ Initial configurations were built with the PACKMOL package^48^ with random atomic positions. Each mixture was equilibrated in NVT conditions for a total time of 6 ns by gradually bringing each system from 300 to 500 K, keeping it at high temperatures for 2 ns and gradually cooling down to 300 K. High-temperature equilibrations were previously observed to be necessary for viscous liquids like DESs and ionic liquids (ILs).^5,49−52^ Production runs for data collection were performed in NVT conditions at 300 K for 50 ns. The temperature was controlled by the Nosé–Hoover thermostat with a relaxation constant of 0.5 ps. The equations of motion were integrated with the leap-frog algorithm (1 fs time step), with trajectories saved every 100 steps. Stretching vibrations involving hydrogen atoms were constrained with the LINCS algorithm.^53^

Site–site radial distribution functions *g*(*r*)’s have been computed for the HBs between MeOH and the DES components ChCl and sesamol, as well as between the DES molecules. The *g*(*r*)’s have been multiplied by the numerical density of the observed atoms (ρ) to properly compare systems with different compositions.^15,54−57^ The coordination numbers *N* have been computed integrating each curve up to a cutoff distance chosen at the position of the first minimum and the obtained values have been reported as a function of *M*.

Simulations were performed with the GROMACS 2020.2 package.^58^ VMD 1.9.3 software^59^ was used for visualization of trajectories.

### SWAXS Measurements and Data Analysis

SWAXS measurements were performed at the SAXSLab Sapienza with a Xeuss 2.0 Q-Xoom system (Xenocs SAS, Grenoble, France), equipped with a microfocus GeniX 3D X-ray source (λ = 1.542 Å), a two-dimensional PILATUS3 R 300 K detector, which can be placed at variable distances from the sample, and an additional PILATUS3 R 100 K detector at fixed shorter distances from the sample to access larger scattering angles (DECTRIS Ltd., Baden, Switzerland). Calibration of the scattering vector *q* range, where *q* = (4πsin θ)/λ, 2θ being the scattering angle, was performed using silver behenate for the SAXS detector position and Al_2_O_3_ for the WAXS detector. Measurements with two sample-SAXS detector distances (550 and 236 mm) were performed so that the overall explored *q* region was 0.02 < *q* < 3.7 Å^–1^. Liquid samples were loaded into disposable borosilicate glass capillaries with a nominal thickness of 1.5 mm and sealed with hot glue before placing them in a vertical position at room temperature (25 ± 1 °C) in the instrument sample chamber, which was then evacuated (≈0.2 mbar). The beam size was defined through the two-pinhole collimation system equipped with “scatterless” slits to be 0.5 mm × 0.5 mm when measuring in the low-*q* regime (0.02 < *q* < 0.6 Å^–1^) and 0.5 mm × 2 mm when measuring in the larger *q* regime (0.03 < *q* < 1.9 Å^–1^). The signal collected by the fixed WAXS detector (1.54 < *q* < 3.7 Å^–1^) in the two exposures was averaged. The two-dimensional scattering patterns were subtracted for the dark counts and then masked, azimuthally averaged, and normalized for transmitted beam intensity, exposure time, and subtended solid angle per pixel using FoxTrot software developed at SOLEIL. The one-dimensional intensity vs *q* profiles was then subtracted for the empty capillary contribution and put in absolute scale units (cm^–1^) by dividing for the capillary thickness estimated by the scans acquired during sample alignment. The different angular ranges were merged using the SAXS Utility tool.^60^

To follow the evolution of the scattering profiles as a function of the increasing MeOH content in the samples, values of the radius of gyration (*R*_g_) and of the intensity extrapolated at a zero angle (*I*(0)) were extracted by performing a linear fit of ln(*I*(*q*)) vs *q*^2^ for 0.06 < *q* < 0.20 Å^–1^, according to the Guinier approximation *I*(*q*) ≈ *I*(0) exp(−*q*^2^*R*_g_^2^/3).

The SWAXS data in the range 0.05 < *q* < 0.8 Å^–1^ were also modeled as an Ornstein–Zernike decay (OZ) with two parameters (the scale *I*_OZ_(0) and the correlation length ξ_OZ_ related to the slope of the intensity decay), plus a background accounting for the rise of the WAXS contribution at *q* > 0.5 Å^–1^, which was described as the sum of two Lorentzian peaks, rather than a flat background. The fitting was performed with SasView software.^61^

Due to the shift of the main WAXS peak position as a function of the composition and its bimodal nature (*vide infra*), the parameters for this background contribution were first optimized for each sample to reproduce the WAXS data (in the range 0.8 < *q* < 1.5 Å^–1^) and then kept fixed in the optimization of the parameters of the OZ contribution. The overall model intensity used and the explicit background contribution are reported in the equations below:
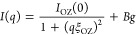
1
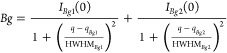
2

The obtained values of ξ_OZ_ can be compared to the *R*_g_ of the Guinier approximation by calculating √3ξ ≈ *R*_g_.

To investigate the origin of the SAXS signal measured in the low-*q* region (<0.5 Å^–1^), theoretical scattering profiles were calculated with the method implemented in CRYSOL^62^ from the atomic coordinates of the sesamol, choline, and chloride species involved in one representative coordination geometry captured by MD simulation of the sample with *M* = 24. The calculation was performed in the range 0 < *q* < 0.5 Å^–1^, with a maximum order of spherical harmonics equal to 15 301 data points, assuming an electron density of the homogeneous solvent either equal to that of pure MeOH (0.269 electrons·Å^–3^) or to that of a MeOH/ChCl/sesamol 24:1:3 mixture (0.312 electrons·Å^–3^) and suppressing the contribution of a solvation shell with different contrast.

## Results and Discussion

### ATR-FTIR Analysis

**Figure 2:**
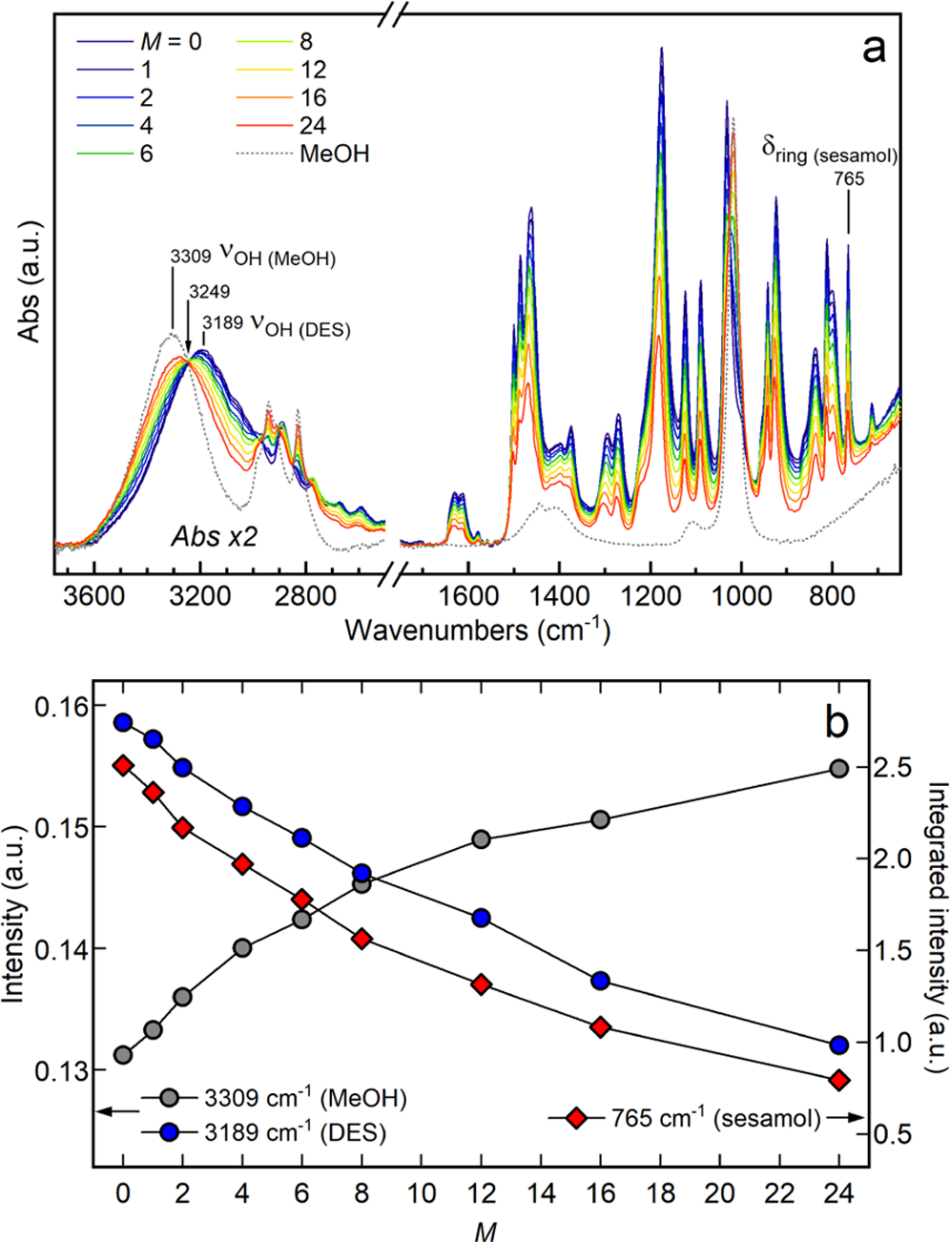
(a) ATR-FTIR spectra of MeOH/ChCl/sesamol mixtures at different *M*:1:3 molar ratios and of pure MeOH. The 3800–2500 cm^–1^ region is enlarged by a factor of 2. (b) Band intensities at 3189 cm^–1^ (blue circles) and 3309 cm^–1^ (gray circles) and integrated intensity at 765 cm^–1^ (red diamonds) as a function of *M*.

ATR-IR spectra were collected on MeOH/ChCl/sesamol mixtures at different *M*:1:3 molar ratios. The obtained data are shown in Figure 2a together with the spectrum collected on pure MeOH. As far as the ChCl/sesamol 1:3 DES (*M* = 0) is concerned, the broad band centered at 3189 cm^–1^ (ν_OH_(DES)) is assigned to the O–H stretching of both ChCl and sesamol hydroxyl groups, which absorb in the same spectral range. These bands were previously observed to be red-shifted with respect to the O–H absorption of the pure components, evidencing the formation of the eutectic through the establishment of strong HBs between the chloride anion with the choline cation and the sesamol molecule.^15,37^ The additional spectral features at lower wavenumbers are assigned exclusively to the sesamol moiety, in particular the C=C stretching at 1610 cm^–1^, the C–H bending region (1500–1300 cm^–1^), the strong C–O stretching absorption between 1300 and 1000 cm^–1^, and the out-of-plane ring deformation (δ_ring_(sesamol)) at 765 cm^–1^.^63^ Unfortunately, bands related to ChCl only are too weak and unresolved to be employed in the analysis.

After MeOH addition to the eutectic, all of the bands related to the ChCl/sesamol 1:3 DES between 1700 and 650 cm^–1^ are found to decrease in intensity, while the MeOH C–O stretching at 1018 cm^–1^ and the −CH_3_ asymmetric and symmetric stretching bands at 2942 and 2831 cm^–1^, respectively, increase. As regards the 3700–3000 cm^–1^ spectral region, the intensity of the ν_OH_(MeOH) band gradually increases at the expense of the ν_OH_(DES) one, approaching the absorption of pure MeOH (ν_OH_(MeOH) = 3309 cm^–1^). As evidenced by the appearance of an isosbestic point at 3249 cm^–1^, the O–H absorption of the MeOH/ChCl/sesamol mixtures can be considered as the weighted sum of the stretching bands of pure MeOH and of the DES.

To retrieve information about the single components, the intensities of the ν_OH_(DES), ν_OH_(MeOH), and δ_ring_(sesamol) bands have been reported as a function of *M* (Figure 2b). As can be observed, the intensities of the analyzed bands are strictly correlated among each other, since the growth of the ν_OH_(MeOH) band corresponds to an equal decrease of the ν_OH_(DES) and δ_ring_(sesamol) absorption upon MeOH addition. Therefore, according to the IR results, the spectroscopic variations of the MeOH/ChCl/sesamol system can be ascribed exclusively to the concentration change of the mixture components. Furthermore, no additional O–H vibrational contributions at different wavenumbers appeared in the spectrum upon MeOH addition. Altogether these results highlight a system that is close to the ideal miscibility, where the average strength of the HB interactions established by MeOH in all of the explored concentration ranges is approximately equal to those observed between the pure components. This result differs from that obtained for mixtures of the ChCl/sesamol 1:3 DES with water, where segregation between sesamol- and water-rich regions was observed, up to the formation of aqueous pools of ∼70 Å diameter for high water molar fractions.^15^ In the framework of the FTIR analysis, the formation of such heterogeneities was detected from the split of the spectral bands connected with distinct water populations: weakly H-bonded water interacting with the DES components absorbing at higher wavenumbers, and a second ensemble of water aggregates showing a network of HBs similar to bulk water. Note that IR studies of nonideal mixtures have been reported also for other DESs^26,64^ and hydrophobic ILs^65−67^ using MeOH as the cosolvent and, in those cases, the formation of imbalanced intermolecular interactions led to the splitting and/or blue shifting of the stretching bands connected to MeOH. Differently, the absence of such phenomena in the absorption spectra of the MeOH/ChCl/sesamol mixtures highlights that the system tends to preserve the same HB average strength across the explored composition range.

### Molecular Dynamics Results

**Figure 3:**
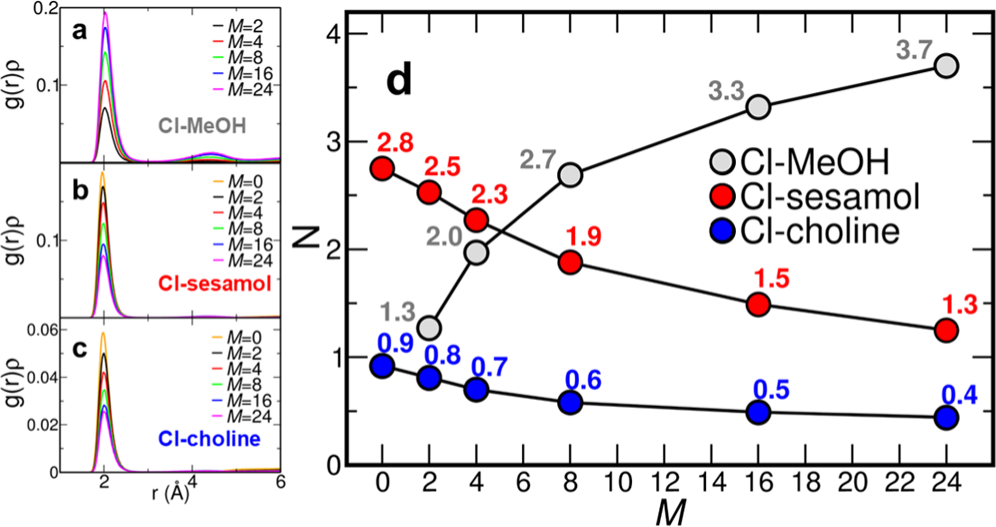
Radial distribution functions multiplied by the numerical densities of the observed atoms, *g*(*r*)ρ’s, calculated between the chloride anion and the hydrogen atom of the hydroxyl group of (a) MeOH, (b) sesamol, and (c) choline from the MD simulations of MeOH/ChCl/sesamol mixtures at different *M*:1:3 molar ratios. (d) Corresponding coordination numbers *N*, taken at the first minimum of the *g*(*r*)ρ’s, plotted as a function of *M*.

To gain further insights into the ChCl/sesamol 1:3 DES structural changes upon MeOH addition, MD simulations have been carried out on MeOH/ChCl/sesamol *M*:1:3 mixtures with *M* = 0, 2, 4, 8, 16, and 24. First, insights into the DES structural arrangement before MeOH addition can be gained by observing the *g*(*r*)’s calculated for *M* = 0. In particular, the interactions involving the chloride anion are shown in Figure 3. In the ChCl/sesamol 1:3 DES, the chloride anion is surrounded by an average number of 2.8 sesamol molecules and 0.9 choline cations coordinating with the hydrogen atom of the hydroxyl moiety (Figure 3d). Differently, the *g*(*r*)’s calculated for the sesamol–sesamol (Figure S1), choline–choline (Figure S2), and choline–sesamol (Figure S3) HBs show *N* values close to zero for *M* = 0, indicating that the interaction between these species is negligible in the pure DES. This picture is in agreement with previous findings describing the structure of this eutectic as formed by discrete chloride clusters that see almost the entire amount of sesamol and choline molecules involved, packed to maximize the HB interactions.^36,37^ When MeOH is added to the DES, the *N* value for the Cl–MeOH interaction (Figure 3a) between the chloride anion and the hydroxyl group of MeOH increases sharply with increasing MeOH concentration (Figure 3d). In detail, an average number of 1.3 MeOH molecules is found in the coordination sphere of the chloride anion for *M* = 2, while the chloride anion is coordinated by 3.7 MeOH molecules for *M* = 24. On the other hand, Cl–sesamol (Figure 3b) and Cl–choline (Figure 3c) interactions show an opposite trend. In particular, both the Cl–sesamol and Cl–choline HBs are found to decrease with increasing MeOH concentration, up to *N* values of, respectively, 1.3 and 0.4 for *M* = 24 (Figure 3d). The evolution of the obtained values shows that when MeOH is added to the ChCl/sesamol 1:3 DES, a favorable interaction with the chloride anion is established so that MeOH is able to displace a considerable amount of sesamol and choline molecules from the chloride anion coordination sphere.

**Figure 4:**
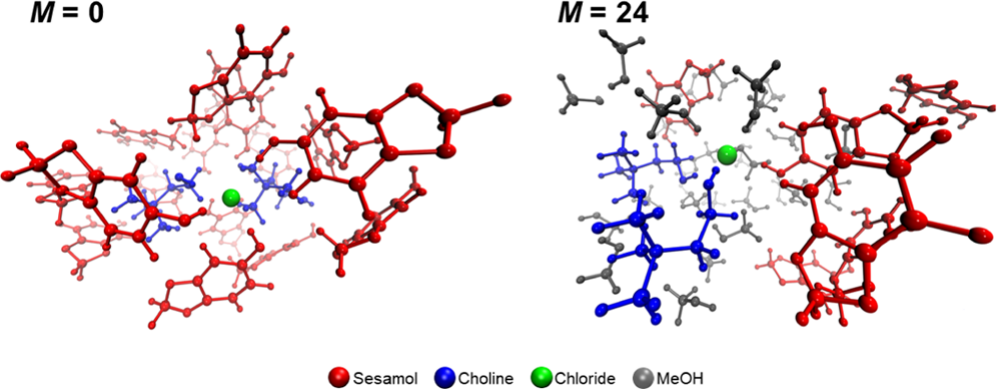
Representative snapshots showing the chloride anion coordination obtained from MD simulations of the MeOH/ChCl/sesamol *M*:1:3 mixtures with *M* = 0 (pure DES, left panel) and *M* = 24 (right panel). The different species are colored according to the color code reported at the bottom. Perspective view and depth cueing are adopted for the sake of clarity.

In addition, it can be noticed that the number of total species interacting with the chloride anion increases with increasing MeOH content, from a total *N* value of 3.7 for *M* = 0 to 5.4 for *M* = 24 (Figure 3d). This is in line with a picture in which the chloride anion tends to maximize the number of HBs with the surrounding species. Note that in the pure ChCl/sesamol 1:3 DES, this value cannot exceed 4 for stoichiometric reasons (three sesamol and one choline molecules interacting *via* the hydroxyl groups). However, in the ChCl/sesamol 1:3 DES, the chloride anion was also found to interact with the two hydrogen atoms of the sesamol dioxolane functionality and with the choline molecule cationic core.^37^ Despite these interactions being weaker with respect to those involving the hydroxyl groups of the components, they also take part in the eutectic structural arrangement, since such interacting sesamol and choline molecules are in turn able to coordinate different chloride anions with the hydroxyl groups and to bridge between the discrete chloride anion clusters.^37^ When MeOH is added to the eutectic, these additional interactions are drastically reduced, as the Cl^–^ anion prefers to form HBs with the MeOH molecules. As an example, the coordination numbers for the interactions of the chloride anion with the two hydrogen atoms of the sesamol dioxolane functionality and with the choline molecule cationic core are 0.9 and 1.7 for *M* = 0, while they are reduced to 0.5 and 1.2 already for *M* = 2, respectively. The local environment around the chloride anion in the two extremes of the explored composition range, *i.e*., *M* = 0 and *M* = 24, is depicted by MD representative snapshots in Figure 4.

The question arising from this picture is what the fate of sesamol and choline molecules is when they are removed from the chloride coordination. To address this issue, one can take a look at the *g*(*r*)’s for the sesamol–sesamol, choline–choline, and choline–sesamol HBs calculated for *M* values in the 2–24 range (Figures S1–S3). As can be observed, the interactions between these species, which were found to be negligible in the pure ChCl/sesamol 1:3 DES, remain insignificant also after MeOH addition, as shown by the *N* values that are still close to zero even for *M* = 24. This result shows that the displacement of sesamol and choline molecules from the chloride anion coordination sphere does not promote a subsequent increase in the interactions between these species. Differently, the sesamol–MeOH HBs show a marked increase upon MeOH addition (Figures S4 and S5), and the same is observed for the choline-MeOH ones (Figures S6 and S7). Furthermore, the HBs formed by MeOH with other MeOH molecules are also found to grow with increasing *M* (Figure S8).

MD results, therefore, highlight that upon MeOH addition, a one-by-one substitution occurs between the HBs that are initially established among the DES components and HBs that see the participation of MeOH. Note that the Cl–MeOH *g*(*r*)’s show well-defined first peaks occurring at comparable distances as compared with the Cl–sesamol and Cl–choline ones (∼2 Å, Figure 3a–c). This points out that the Cl–sesamol and Cl–choline HBs are replaced by the Cl–MeOH ones that are similar in strength. In the meanwhile, the displaced sesamol and choline molecules are solvated by MeOH, so that the system arranges itself to keep the overall amount of HB interactions constant. This picture confirms the observation of the FTIR analysis showing that no substantial variation in the overall average strength of the HBs occurs through the explored composition range. This behavior is different from what was previously observed for water/ChCl/sesamol *W*:1:3 mixtures, where the segregation between sesamol- and water-rich regions was promoted by the saturation of the sesamol–water HBs after the *W* = 6–8 threshold.^15^ Differently, the ability of MeOH to interplay with all of the DES components through all of the explored molar ratios is at the basis of the observation of no segregation effects in the studied MeOH/ChCl/sesamol mixtures. Such results can also be observed at a more qualitative level from the snapshots of the simulated MD systems in Figure S9.

### SWAXS Analysis

**Figure 5:**
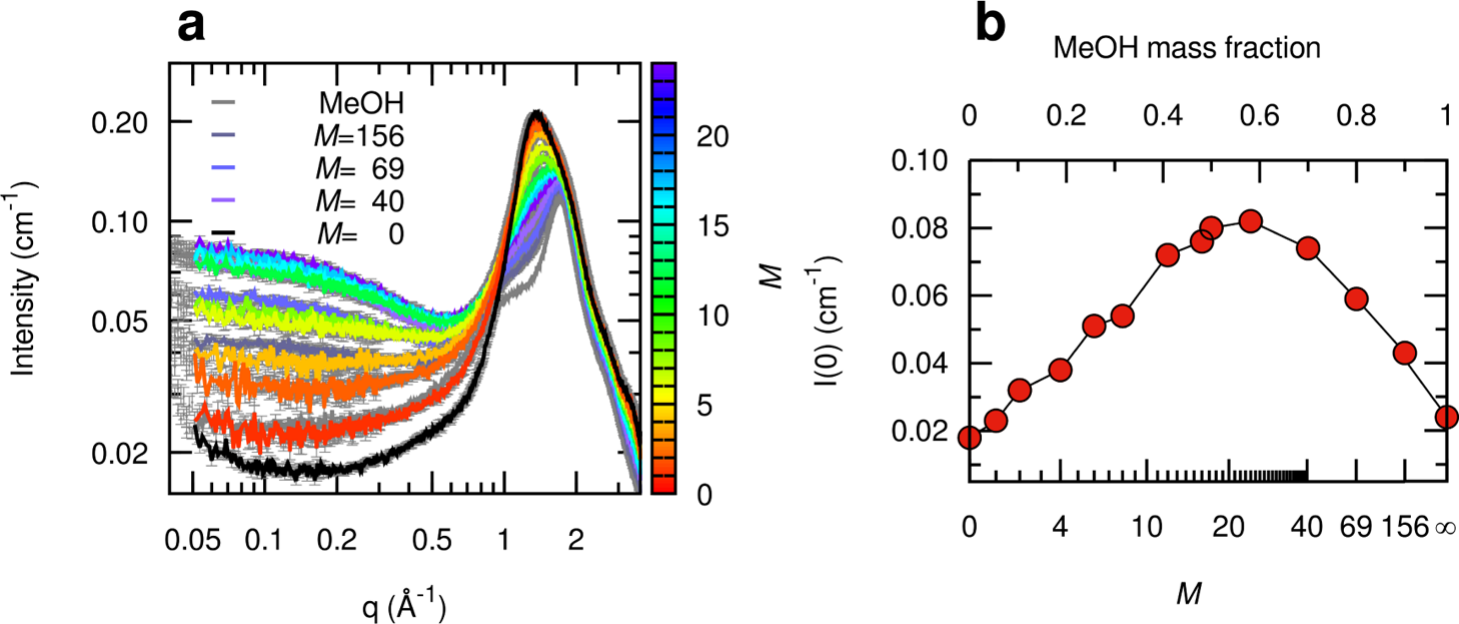
SWAXS characterization of MeOH/ChCl/sesamol mixtures at different *M*:1:3 molar ratios (0 ≤ *M* ≤ 156). (a) Experimental scattering profiles are shown as solid lines with a color code related to *M* (color bar 0 < *M* ≤ 24, and key for *M* = 0, 40, 69, 156, and ∞, *i.e*., pure MeOH). (b) Scattered intensity extrapolated at zero angles as a function of *M*. The corresponding sample composition as MeOH mass fraction is also reported in the upper linear scale.

SWAXS data were first collected on MeOH/ChCl/sesamol *M*:1:3 mixtures with *M* = 0–24 (Figure 5a, data according to the color bar). In the small-angle region (*q* < 0.5 Å^–1^), an increase in the scattered intensity with increasing *M* values was observed. To better investigate the origin of this growth and to explore the effect of the introduction of MeOH at higher molar ratios, additional samples were analyzed in the *M* = 40–156 range (Figure 5a, data according to the key). Note that the introduction of such high MeOH content did not provoke any phase separation, differently from what was observed for water/ChCl/sesamol *W*:1:3 mixtures after *W* = 20–26.^15^ The collected spectra show that the effect reaches a maximum for *M* = 24 and then starts to decrease toward the scattering signal of pure MeOH, as can also be observed from the values of the intensity extrapolated at zero angles (Figure 5b). At the same time, the WAXS main peak moves from *q* = 1.36 Å^–1^ in the pure DES^37^ to *q* = 1.72 Å^–1^, where the main WAXS peak of pure MeOH is found (Figure S10). It can be noticed, however, that the broad WAXS peaks of the mixtures subtend at least two contributions, the former at lower *q* (related to the characteristic distances of the pure DES) and the latter at *q* larger than 1.5 Å^–1^ becoming increasingly relevant with increasing MeOH content. When reporting only the position of the absolute WAXS maximum as a function of composition (Figure S10b), an apparent abrupt shift is seen between *M* = 8 (MeOH mass fraction 0.32, approximate volume fraction 0.43) and *M* = 12 (MeOH mass fraction 0.42, approx. volume fraction 0.52) due to the inversion of the order of relative intensity of the two contributions. Similar to what has been observed from the analysis of the ATR-FTIR data, the WAXS peak evolution as a function of the MeOH content simply follows the volume fraction change of the mixture components.

**Figure 6:**
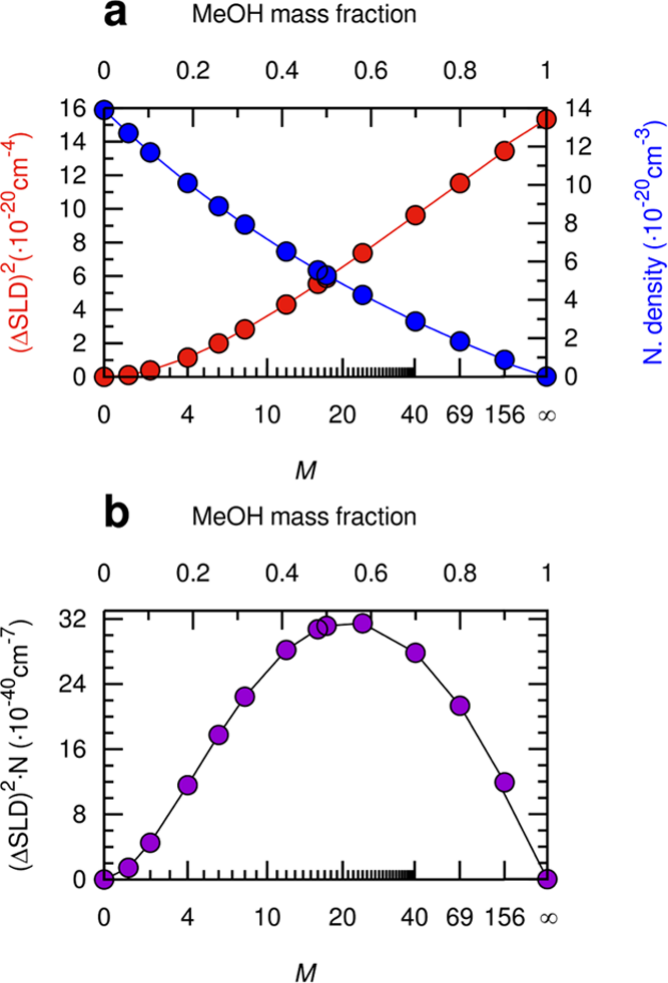
(a) Estimates of the square of the scattering length density contrast (ΔSLD)^2^ (red circles, left vertical axis) and the number density of the chloride anions (blue circles, right vertical axis) in MeOH/ChCl/sesamol mixtures at different *M*:1:3 molar ratios as a function of *M* and MeOH mass fraction (upper linear scale). The ΔSLD is calculated between the pure ChCl/sesamol 1:3 DES (density 1.2791 g mL^–1^, electron density 0.408 electrons·Å^–3^) and a background having the average electron density of MeOH/ChCl/sesamol *M*:1:3 mixtures (from 0.396 electrons·Å^–3^ for *M* = 1 to 0.278 electrons·Å^–3^ for *M* = 156), obtained assuming ideality of the mixtures and the MeOH density equal to 0.7863 g mL^–1^. (b) Product of the (ΔSLD)^2^ and of the chloride anion number density as a function of *M* gives an estimate for the expected trend of SAXS signal originating from electron density correlations of constant volume. Within the simplified framework of a monodisperse and ideal solution of particles (*i.e*., inhomogeneities of electron density), the SAXS intensity extrapolated at zero angles in absolute units would be given by further multiplying the contrast factor and the number density by the volume of the particle.

The presence of a maximum in the MeOH concentration dependence of the SAXS intensity (Figure 5b) testifies the existence of electron density fluctuations, with a size that can be estimated from the slope of the low-*q* intensity decay (*vide infra*) and whose contribution is maximized for the *M* = 24 composition. We can hypothesize that the electron density fluctuations giving rise to the SAXS signal originate from the existence of coordination clusters around the chloride anion, as indicated by the MD simulations, which can represent regions with a higher electron density (close to that of the pure DES, 0.408 electrons·Å^–3^) as compared to a progressively more MeOH-rich background. Assuming for simplicity that there is no variation of the volume of such inhomogeneities upon MeOH addition, the possible evolution of their scattered intensity as a function of the composition can be estimated from the product of the square of their scattering length density contrast (ΔSLD)^2^, which should increase as the average electron density decreases due to MeOH addition, and their number concentration, which would fall off according to the diminishing DES volume fraction in the mixture (Figure 6). Such a simplified assumption predicts a maximum for the SAXS signal for compositions of *M* = 20–24, in agreement with the experimental observations. This supports the hypothesis that in the MeOH/ChCl/sesamol *M*:1:3 mixtures, the same spatial correlations existing in the pure DES and ruled by the interaction with the chloride anion take place, but they are progressively diluted in a solvent background, which becomes richer in MeOH and therefore progressively less electron-dense.

**Figure 7:**
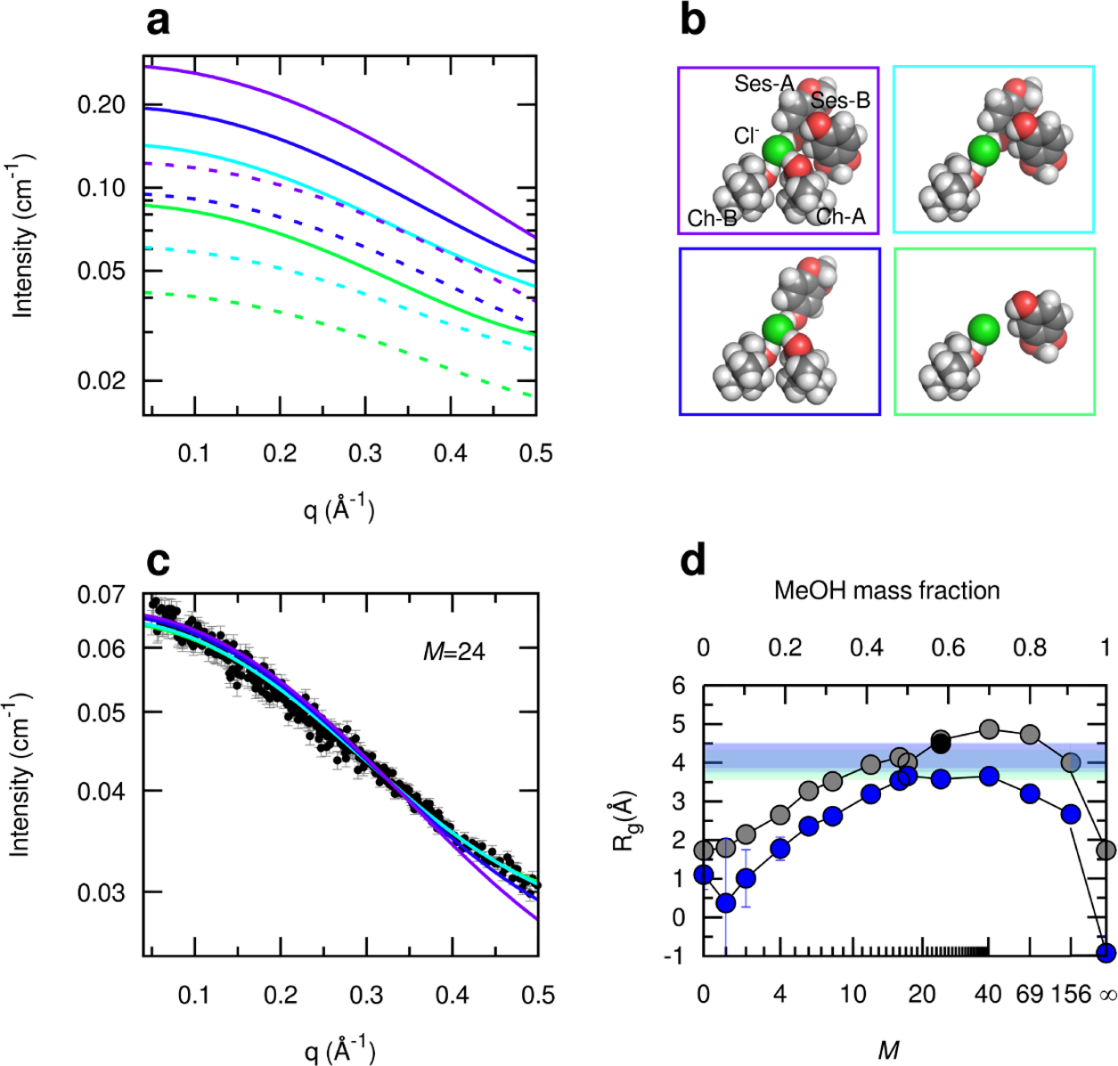
(a) Theoretical scattering profiles calculated using CRYSOL^62^ from the atomic coordinates of the sesamol, choline molecules, and chloride anion species involved in one representative coordination geometry captured by the MD simulation of the sample with *M* = 24 (Figure 4), from which the MeOH molecules were removed since assumed as part of a background with lower homogeneous electron density. The solid lines refer to the calculated intensity assuming a homogeneous background with the electron density of pure MeOH, whereas the dashed lines refer to the theoretical profile assuming a background electron density equal to that of a MeOH/ChCl/sesamol 24:1:3 mixture. The color code refers to the different assemblies shown in panel b, corresponding to the full coordination of the chloride anion by non-MeOH species as found in the MD trajectory frame (purple, two choline ions Ch-A and Ch-B, and two sesamol molecules Ses-A and Ses-B) or smaller clusters with only three (cyan, Ch-B, Ses-A, and Ses-B, or blue, Ch-A, Ch-B, and Ses-A) or two (green, Ch-B and Ses-B) non-MeOH molecules around the chloride anion, which also gave theoretical scattering profiles close to the experimental ones. In panel c, the theoretical profiles are fitted through a scaling constant and a residual flat background to the experimental data collected for *M* = 24, subtracted for the experimental pure MeOH contribution scaled for a volume fraction of 0.69 (black dots). In panel d, the experimental values of the radius of gyration *R*_g_ from the Guinier fit (blue circles) or calculated as √3ξ_OZ_, where ξ_OZ_ is the best fit correlation length of the Ornstein–Zernike function (gray circles, listed in Table S3), are compared to the values obtained from the theoretical intensity profiles of panel a, shown as colored belts having as lower and upper limiting values those calculated assuming solvent electron density equal to the average of the *M* = 24 composition, and of pure MeOH, respectively. The black circle represents the *R*_g_ obtained by Guinier fit of the MeOH-subtracted data shown in panel c.

More quantitative characterization of the system structural properties can be obtained by calculating the radius of gyration *R*_g_ from the slope of the SAXS intensity. Indeed, *R*_g_ can be thought of as the analogue of the radius of gyration of distribution of masses in mechanics, but weighted by the electron density rather than the mass and it is known to be indicative of the three-dimensional size of a particle, a macromolecule, or a molecular aggregate in solution surrounded by a background electron density. In a complex fluid, it can be taken as an estimate of the spatial extension of electron density fluctuations in the system.^34,68,69^ This was done both by applying the Guinier approximation for *q* < 0.2 Å^–1^ to obtain a value of the radius of gyration *R*_g_ and by performing a fit of the data (*q* < 0.8 Å^–1^) according to an Ornstein–Zernike decay plus a background (Figure S11), providing a value of the correlation length ξ_OZ_, which can be compared to the *R*_g_ of the Guinier approximation by calculating √3ξ ≈ *R*_g_ (Figure 7d). The evaluation of *R*_g_, considering the lengthscale close to the molecular size, is partially affected by the choice of the background to be considered: in the case of the *M* = 24 mixture, which showed the highest SAXS intensity, a value of 3.5 Å is obtained from the Guinier fit of the raw data, whereas a value of 4.5 Å is obtained from the Guinier fit of the data subtracted for the experimental pure MeOH contribution scaled for a volume fraction of 0.69. The *R*_g_ value obtained as √3ξ from the fit with an Ornstein–Zernike decay plus a background is in agreement with a value of 4.5 Å.

To further investigate the origin of the SAXS signal, we also calculated the theoretical scattering profiles of small clusters of cholinium and sesamol molecules coordinating the chloride anion as obtained in the MD simulation (Figure 7a,b). We considered a MD snapshot with a relatively high coordination of the chloride anion by non-MeOH molecules (two sesamol molecules and two cholinium ions), from which the MeOH molecules were removed since in this simplified view they are assumed as part of a background with lower homogeneous electron density. All of the possible molecular assemblies with a lower number of non-MeOH coordinating molecules around the chloride anion were also obtained from these configurations by removing selected cholinium and sesamol molecules. The calculated scattering profiles that were more in agreement with the experimental SAXS data of the *M* = 24 mixture (Figure 7c) included those of assemblies with all four and also three or two non-MeOH molecules around the chloride anion. They corresponded to slopes with *R*_g_ between 3.5 and 4.5 Å, depending on whether the electron density of the homogeneous background was imposed as the average of the MeOH/ChCl/sesamol 24:1:3 mixture or of pure MeOH, respectively, considering that the selected clusters had a calculated electron density between 0.393 and 0.411 electrons·Å^–3^, higher than the background and close to that of the pure DES. This comparison with the simulated SAXS profiles supports the interpretation that the measured scattering signal arises from an intrinsic electron density contrast at molecular lengthscales within the first coordination sphere of the chloride anion.

It should be mentioned that the highest SAXS intensity detected for the MeOH/ChCl/sesamol mixtures (*M* = 24) was less than half of that measured for a water/ChCl/sesamol mixture with 16:1:3 molar ratios, which has been found to be the macroscopically homogeneous water/DES mixture with the highest water content that can be prepared before phase separation.^15^ Considering also that for the water/ChCl/sesamol 16:1:3 mixture the corresponding estimate for √3ξ from the SAXS data was 29 Å, it is understandable by comparison that in the MeOH/ChCl/sesamol system a phenomenon of segregation at the nanoscale level does not occur at any composition. Indeed, as previously mentioned, *R*_g_ can be considered as a rough approximation of the biggest aggregate dimensions, and the values obtained for the present mixtures are not compatible with nanoscale aggregations, but rather with small clusters governed by short-range order interactions. This behavior more closely resembles that observed for aqueous mixtures of other DESs like the ChCl/urea and ChCl/glycolic acid ones, where homogeneity at the nanoscale level was maintained due to the ability of interspersed water to enter the local cluster formed by the chloride anion.^14,16^

## Conclusions

The structural changes of the ChCl/sesamol 1:3 DES upon MeOH addition have been studied. ATR-FTIR spectra collected on MeOH/ChCl/sesamol mixtures at *M*:1:3 molar ratios in the *M* = 0–24 range show that the band related to the MeOH O-H stretching increases in intensity upon MeOH addition, while the intensity of the bands associated to the ChCl/sesamol 1:3 DES components decreases. The appearance of no additional spectral features and the absence of band shifts highlight that the overall strength of the HB interactions between the mixture components is preserved across the explored composition range. MD simulations performed on the same systems show that when MeOH is added to the ChCl/sesamol 1:3 DES, it is able to replace sesamol and choline molecules from the chloride anion coordination sphere through the establishment of Cl–MeOH interactions. This effect does not promote the sesamol–sesamol, choline–choline, and sesamol–choline HBs, which remain as negligible as in the pure DES. Differently, the displaced sesamol and choline molecules are solvated by MeOH, which also forms HBs with other MeOH molecules. The one-by-one substitution of the HBs present in the pure DES with HBs where MeOH replaces the DES components explains the conservation of the overall HBs strength in the explored composition range. SWAXS measurements show that this effect is predominant up to *M* = 20–24, while afterward (*M* = 40–156) the scattering profile is progressively diluted in the cosolvent background and decreases toward the signal of pure MeOH. The ability of MeOH to interact with all of the DES components, as well as with the other MeOH molecules, is at the basis of the observation of neither phase separation nor inhomogeneities at the nanoscale level in the studied mixtures even for high MeOH contents. This behavior is very different from what was previously found for mixtures of the ChCl/sesamol 1:3 DES with water, where pseudophase segregation between sesamol- and water-rich regions was observed. The results here obtained have important implications for the application of DESs in extraction procedures since these solvents are often employed in liquid–liquid microextractions in the presence of dispersing agents, among which alcohols of various chain lengths are ideal candidates. The structural changes that the ChCl/sesamol 1:3 eutectic undergoes upon addition of the prototypical alcohol MeOH can therefore help in addressing the correct target species and experimental conditions for the extraction, while opening the question about the interaction of this quasihydrophobic DES with higher alcohols.

## Supporting Information Available

Additional details of the prepared samples and of the simulated MD systems; additional MD results; and SAXS data visualization and analysis (PDF)
