# Supplementary material for: Fate of a Deep Eutectic Solvent upon Cosolvent Addition: Choline Chloride–Sesamol 1:3 Mixtures with Methanol
Source: ACS Sustain Chem Eng. 2021 Sep 2;9(36):12252–61. doi: 10.1021/acssuschemeng.1c03809 (PMC8442355; doi:10.1021/acssuschemeng.1c03809)
Supplement: Supplementary file 1 — sc1c03809_si_001.pdf [file sc1c03809_si_001.pdf]

Supporting Information

Fate of a Deep Eutectic Solvent upon Co-solvent Addition: Choline Chloride:Sesamol 1:3 Mixtures with Methanol

Matteo Busato, Alessandra Del Giudice, Valerio Di Lisio, Pierpaolo Tomai, Valentina Migliorati, Alessandra Gentili, Andrea Martinelli, and Paola D'Angelo*[a]

[a]Department of Chemistry, University of Rome "La Sapienza", P.le A. Moro 5, 00185, Rome, Italy

*Corresponding author

E-mail: p.dangelo@uniroma1.it

Pages: S1 – S11

Tables: S1 – S3

Figures: S1 – S11

Table S1. List of prepared samples for the MeOH/DES mixtures with MeOH:ChCl:sesamol *M*:1:3 molar ratios.

M	MeOH mass fraction	Density (g cm^{-3})†
0	0.00	1.28
1	0.05	1.24
2	0.10	(1.20)
4	0.19	1.16
6	0.26	(1.10)
8	0.32	1.06
12	0.41	1.04
16	0.48	1.00
17	0.50	(0.98)
20	0.54	0.97
24	0.58	(0.94)
40	0.70	(0.89)
69	0.80	(0.85)
156	0.90	(0.82)

†Experimental densities obtained by weighting 1 mL of mixture in a volumetric flask, while values in parentheses have been calculated assuming ideality from the molar composition as $\boldsymbol{d_M} = \frac{1 \cdot MW_{ChCl} + 3 \cdot MW_{sesamol} + M \cdot MW_{MeOH}}{\frac{1 \cdot MW_{ChCl} + 3 \cdot MW_{sesamol}}{d_{DES}} + \frac{M \cdot MW_{MeOH}}{d_{MeOH}}}$, where $\boldsymbol{d_{DES}}$ = 1.2791 g·cm^{-3} is the density of the ChCl:sesamol 1:3 DES and $\boldsymbol{d_{MeOH}}$ = 0.7863 g·cm^{-3} is MeOH density.

Table S2. Number of molecules and box dimensions of the simulated MD systems for MeOH:ChCl:sesamol mixtures at *M*:1:3 molar ratios.

M	MeOH	ChCl	Sesamol	Box edge (Å)
0	0	1390	4170	99.99
2	2338	1169	3507	99.99
4	4024	1006	3018	99.99
8	6176	772	2316	99.02
16	8848	553	1659	99.98
24	10272	428	1284	100.01

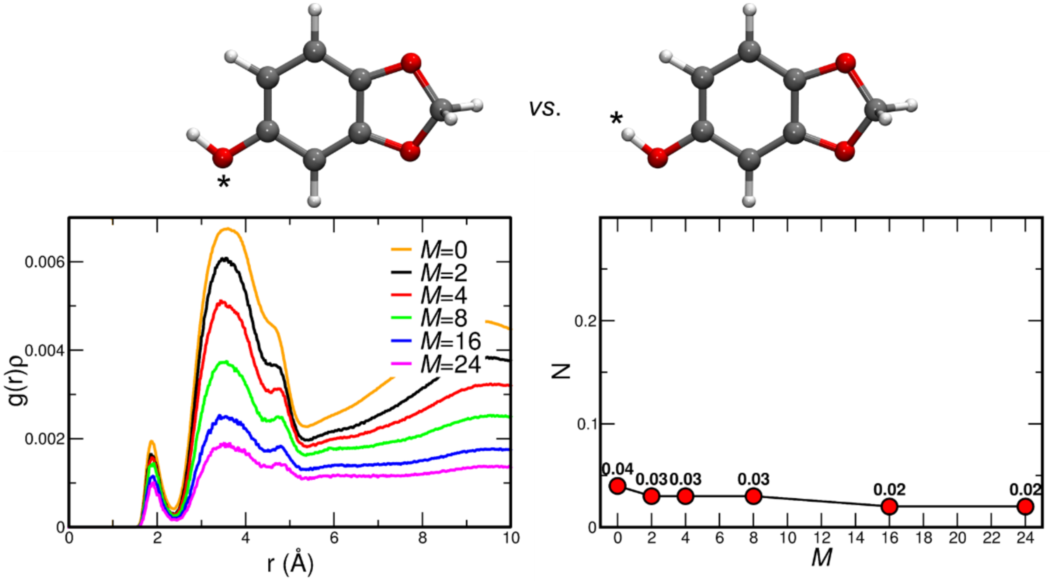

Figure S1. Sesamol–sesamol radial distribution functions multiplied by the numerical densities of the observed atoms, *g(r)ρ's*, calculated from the MD simulations of the MeOH:ChCl:sesamol mixtures at *M*:1:3 molar ratios with *M* = 0, 2, 4, 8, 16 and 24 (left panel) within the coordination numbers *N* of the observed atoms as a function of *M* (right panel). The reference and observed atoms of the *g(r)'s* are indicated with an asterisk on the correspondent molecular structures reported on top.

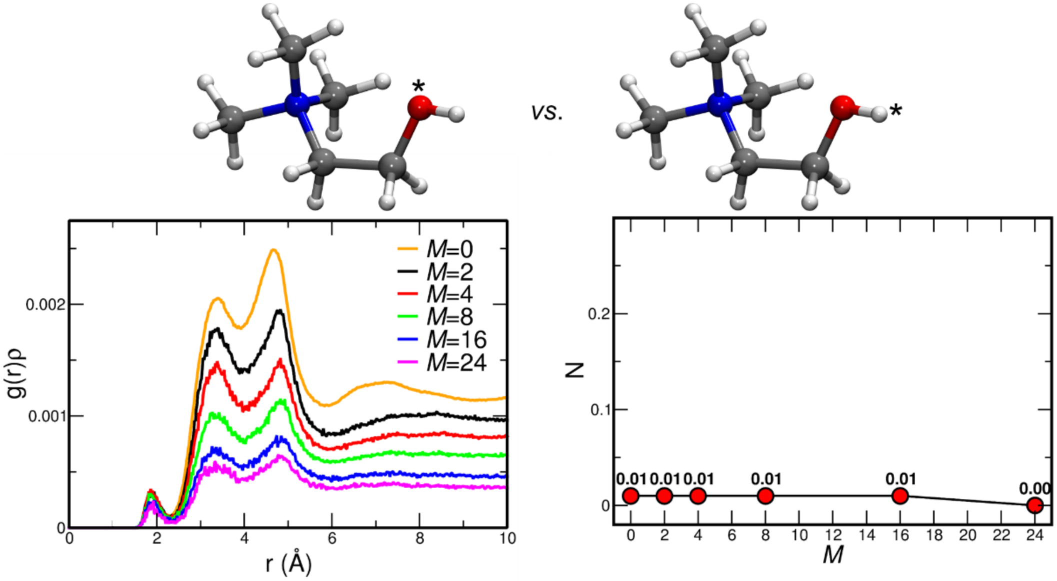

Figure S2. Choline–choline radial distribution functions multiplied by the numerical densities of the observed atoms, *g(r)ρ's*, calculated from the MD simulations of the MeOH:ChCl:sesamol mixtures at *M*:1:3 molar ratios with *M* = 0, 2, 4, 8, 16 and 24 (left panel) within the coordination numbers *N* of the observed atoms as a function of *M* (right panel). The reference and observed atoms of the *g(r)'s* are indicated with an asterisk on the correspondent molecular structures reported on top.

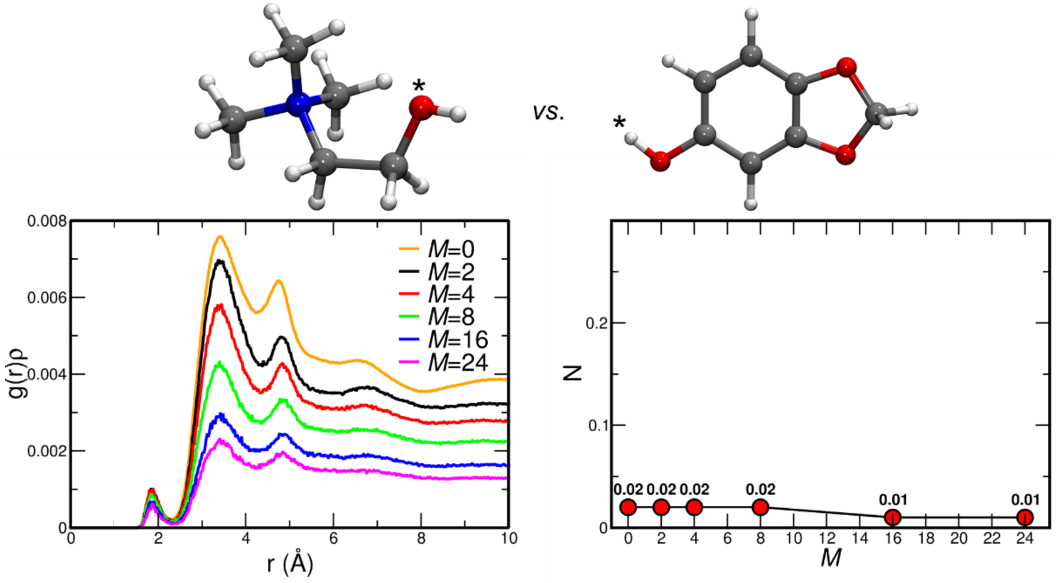

Figure S3. Choline–sesamol radial distribution functions multiplied by the numerical densities of the observed atoms, *g(r)ρ's*, calculated from the MD simulations of the MeOH:ChCl:sesamol mixtures at *M*:1:3 molar ratios with *M* = 0, 2, 4, 8, 16 and 24 (left panel) within the coordination numbers *N* of the observed atoms as a function of *M* (right panel). The reference and observed atoms of the *g(r)'s* are indicated with an asterisk on the correspondent molecular structures reported on top.

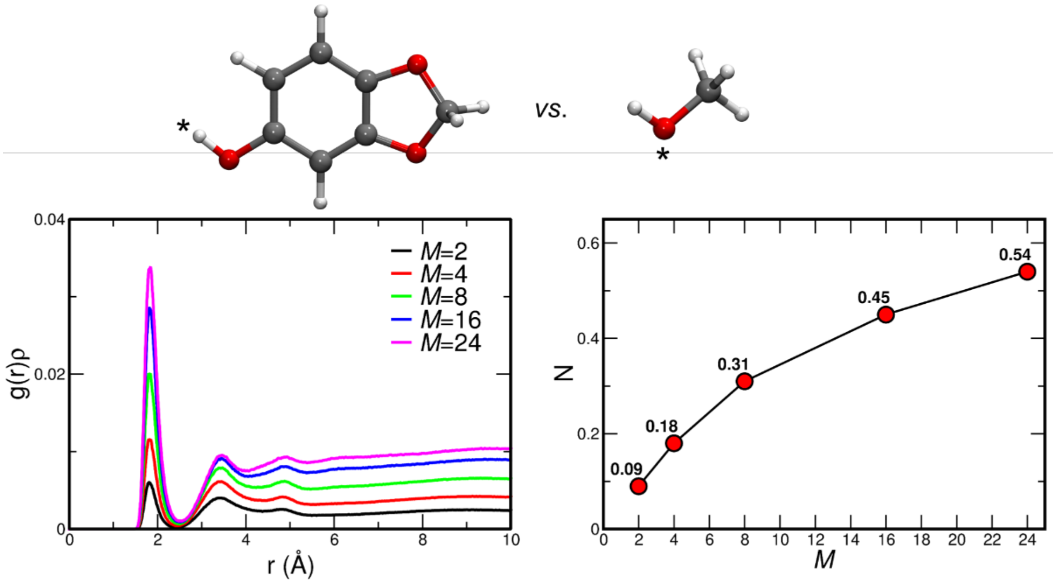

Figure S4. Sesamol–methanol radial distribution functions multiplied by the numerical densities of the observed atoms, *g(r)ρ's*, calculated from the MD simulations of the MeOH:ChCl:sesamol mixtures at *M*:1:3 molar ratios with *M* = 2, 4, 8, 16 and 24 (left panel) within the coordination numbers *N* of the observed atoms as a function of *M* (right panel). The reference and observed atoms of the *g(r)'s* are indicated with an asterisk on the correspondent molecular structures reported on top.

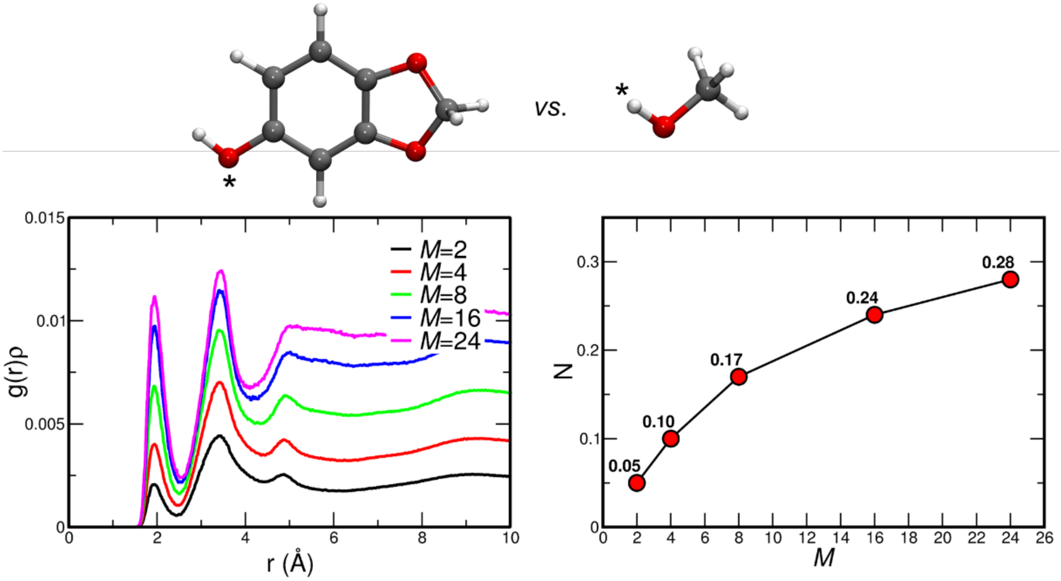

Figure S5. Sesamol–methanol radial distribution functions multiplied by the numerical densities of the observed atoms, *g(r)ρ's*, calculated from the MD simulations of the MeOH:ChCl:sesamol mixtures at *M*:1:3 molar ratios with *M* = 2, 4, 8, 16 and 24 (left panel) within the coordination numbers *N* of the observed atoms as a function of *M* (right panel). The reference and observed atoms of the *g(r)'s* are indicated with an asterisk on the correspondent molecular structures reported on top.

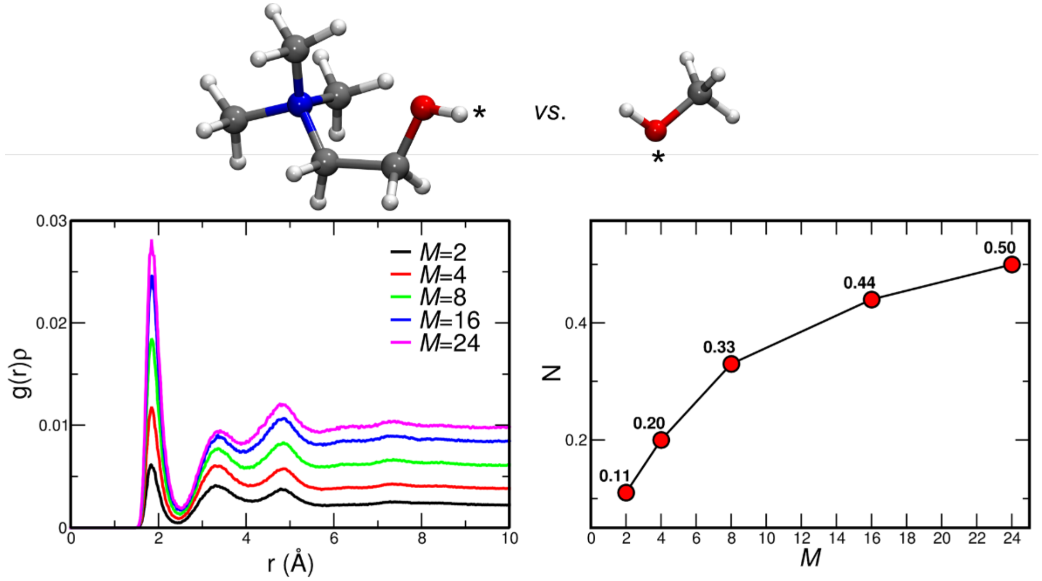

Figure S6. Choline–methanol radial distribution functions multiplied by the numerical densities of the observed atoms, *g(r)ρ's*, calculated from the MD simulations of the MeOH:ChCl:sesamol mixtures at *M*:1:3 molar ratios with *M* = 2, 4, 8, 16 and 24 (left panel) within the coordination numbers *N* of the observed atoms as a function of *M* (right panel). The reference and observed atoms of the *g(r)'s* are indicated with an asterisk on the correspondent molecular structures reported on top.

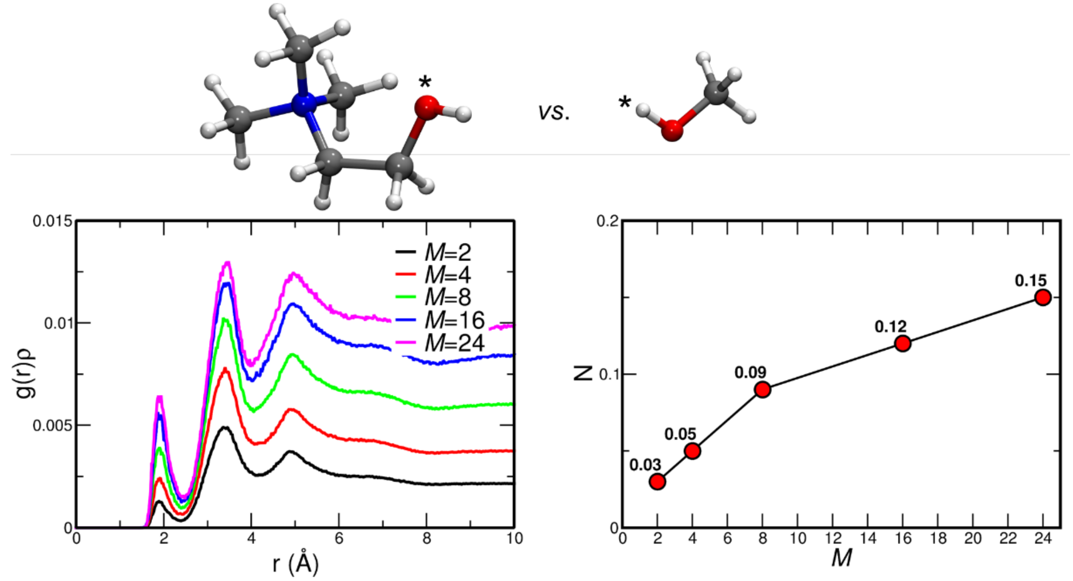

Figure S7. Choline–methanol radial distribution functions multiplied by the numerical densities of the observed atoms, *g(r)ρ's*, calculated from the MD simulations of the MeOH:ChCl:sesamol mixtures at *M*:1:3 molar ratios with *M* = 2, 4, 8, 16 and 24 (left panel) within the coordination numbers *N* of the observed atoms as a function of *M* (right panel). The reference and observed atoms of the *g(r)'s* are indicated with an asterisk on the correspondent molecular structures reported on top.

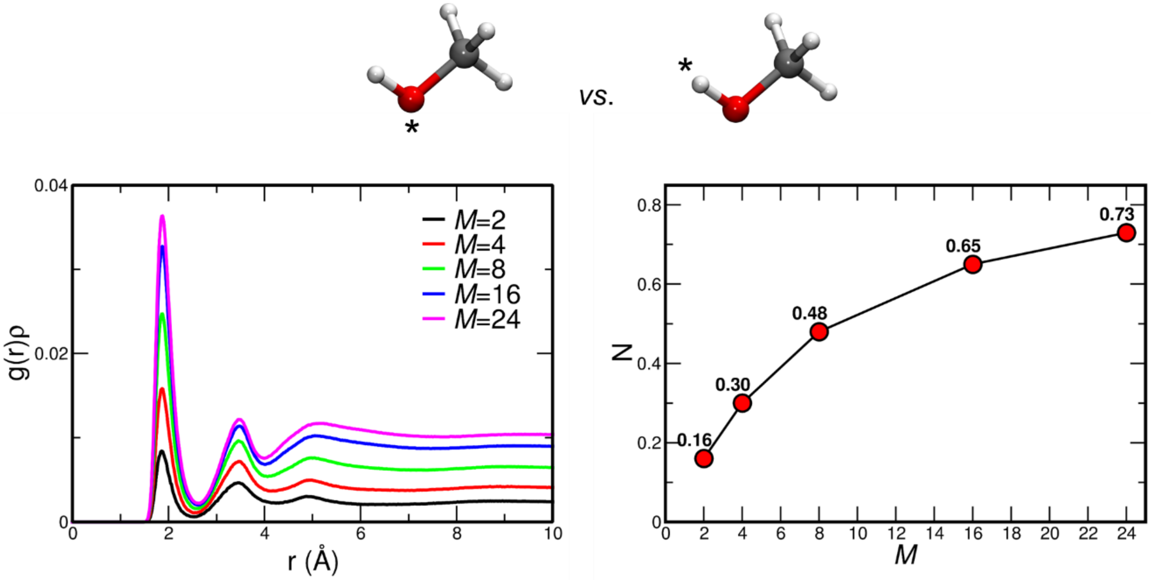

Figure S8. Methanol–methanol radial distribution functions multiplied by the numerical densities of the observed atoms, *g(r)ρ's*, calculated from the MD simulations of the MeOH:ChCl:sesamol mixtures at *M*:1:3 molar ratios with *M* = 2, 4, 8, 16 and 24 (left panel) within the coordination numbers *N* of the observed atoms as a function of *M* (right panel). The reference and observed atoms of the *g(r)'s* are indicated with an asterisk on the correspondent molecular structures reported on top.

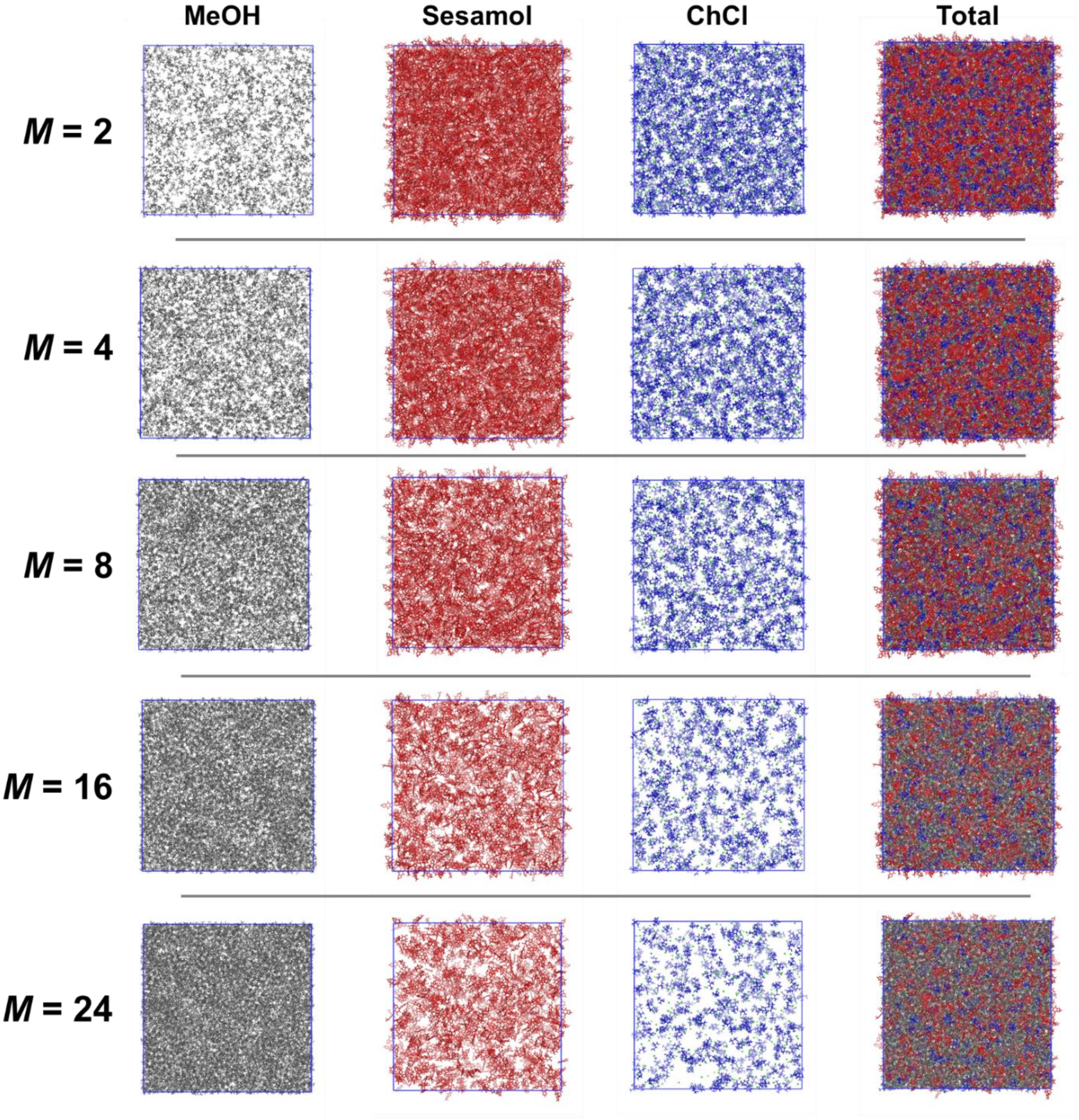

Figure S9. Snapshots taken from the final configuration of the MD simulations performed on MeOH:ChCl:sesamol mixtures at *M*:1:3 molar ratios for *M* = 2, 4, 8, 16 and 24. Single species are shown both alone and together in the corresponding total systems (gray: methanol, red: sesamol, blue: choline, green: chloride). Box edges are highlighted by blue lines.

Table S3. Parameters obtained by the modelling of the experimental SWAXS intensity profiles as an Ornstein-Zernike decay plus a WAXS background given by two Lorentzian peaks, as stated in the Materials and Methods section of the manuscript. An estimate of the uncertainty on the last reported digit is shown in brackets. If not shown, the parameter has been kept fixed in the fitting.

M	$I_{OZ}(0)$ (cm⁻¹)	ξ_{OZ} (Å)	I_{Bg1}(cm⁻¹)	q_{Bg1} (Å⁻¹)	$HWHM_{Bg1}$ (Å⁻¹)	I_{Bg2} (cm⁻¹)	q_{Bg2} (Å⁻¹)	$HWHM_{Bg2}$ (Å⁻¹)
**0**	0.0101(1)	0.89(2)	-	-	-	0.21	1.36	0.23
**1**	0.0139(1)	1.04(2)	-	-	-	0.20	1.36	0.27
**2**	0.0203(1)	1.24(2)	-	-	-	0.19	1.38	0.31
**4**	0.0266(1)	1.53(1)	-	-	-	0.18	1.40	0.34
**6**	0.0375(2)	1.89(1)	-	-	-	0.16	1.44	0.40
**8**	0.0401(2)	2.03(1)	-	-	-	0.15	1.46	0.43
**12**	0.0592(2)	2.28(1)	0.074	1.30	0.39	0.10	1.72	0.40
**16**	0.0624(2)	2.39(1)	0.074	1.30	0.39	0.10	1.72	0.40
**17**	0.0651(2)	2.31(1)	0.063	1.30	0.41	0.10	1.72	0.40
**24**	0.0694(2)	2.65(1)	0.056	1.23	0.46	0.11	1.72	0.40
**40**	0.0610(2)	2.81(1)	0.047	1.18	0.52	0.11	1.72	0.40
**69**	0.0461(1)	2.73(1)	0.041	1.10	0.52	0.11	1.72	0.40
**156**	0.0305(1)	2.31(1)	0.035	1.02	0.44	0.11	1.72	0.40
**100**	0.0132(1)	0.99(2)	0.033	1.01	0.41	0.10	1.74	0.34

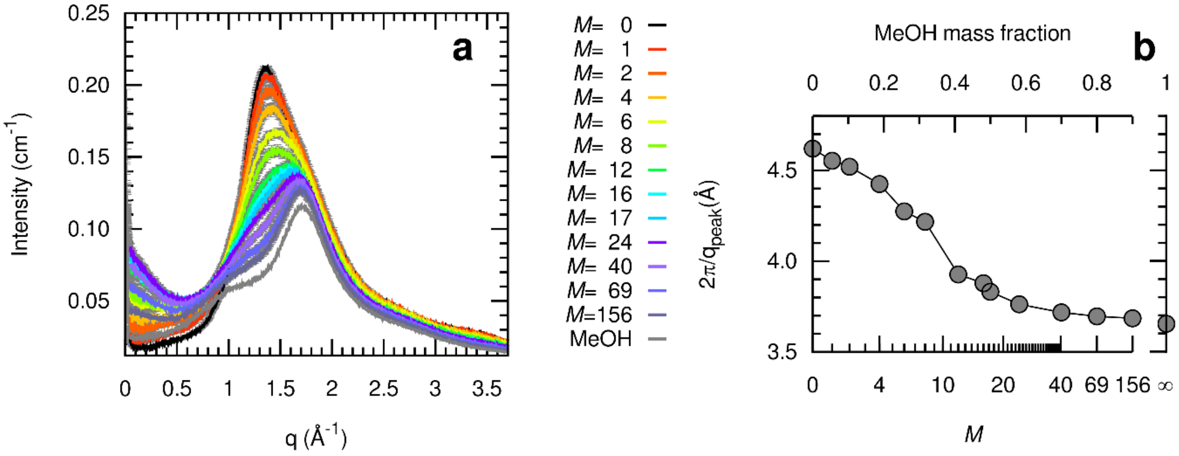

Figure S10. (a) Experimental SWAXS profiles collected on the pure ChCl:sesamol 1:3 DES, on the MeOH:ChCl:sesamol mixtures with different *M*:1:3 molar ratios and on pure MeOH plotted in linear scale to highlight changes of the WAXS peak. (b) Characteristic distance related to the main WAXS peak position (q_{peak}) as a function of *M* and the corresponding MeOH mass fraction (upper linear scale). The broad WAXS peaks subtend at least two contributions, one at lower q (around 1.4 $Å^{-1}$ for the pure DES) and a second at larger q (around 1.6 $Å^{-1}$), resulting in a main maximum and a shoulder. Only the position of the absolute maximum is reported, and it shifts abruptly between M = 8 (MeOH mass fraction 0.32, approx. volume fraction 0.43) and M = 12 (MeOH mass fraction 0.42, approx. volume fraction 0.52) due to the inversion of the order of relative intensity of the two contributions.

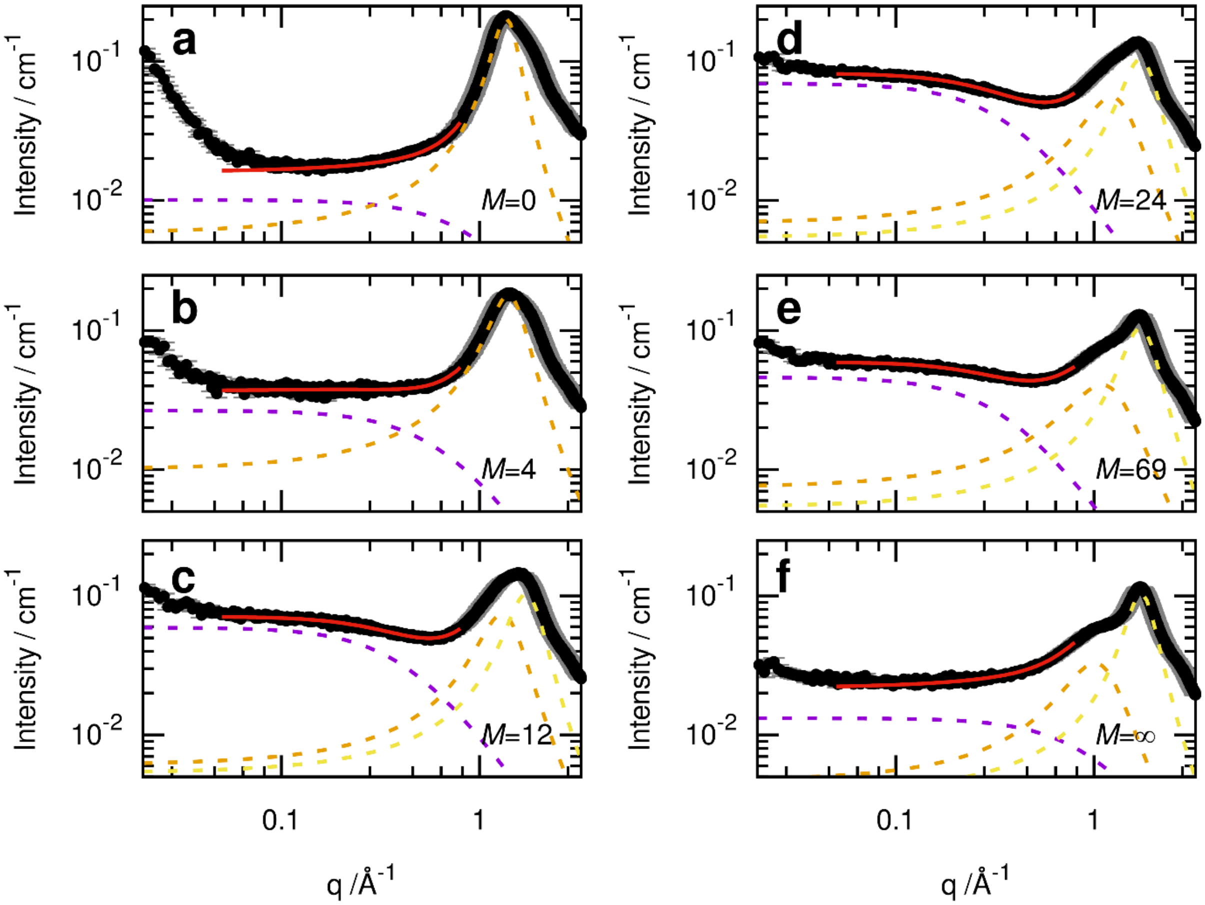

Figure S11. Experimental X-ray scattering profiles (black dots) for some of the studied MeOH:ChCl:sesamol mixtures at different *M*:1:3 molar ratios (*M* = 0, 4, 12, 24, 69, ∞ in panels a, b, c, d, e, f, respectively) compared to the best-fit intensity (solid red lines) according to the sum of an Ornstein-Zernike contribution (OZ, dashed purple lines) and a background contribution also accounting for the rise of the WAXS peak, modelled as a single Lorentzian function (dashed orange lines) or the sum of two Lorentzian functions (the second shown as dashed yellow lines). The model parameters can be found in Table S3.
